# In-vivo tissue healing mechanism at the intestinal anastomosis site following high-frequency electric welding

**DOI:** 10.1097/JS9.0000000000002093

**Published:** 2024-10-11

**Authors:** Caihui Zhu, Yuyan Na, Zhengqing Yan, Xiujun Cheng, Pengyao Xie, Xiaonan Tao, Lei Chen, Hui Zhao, Jian Qiu, Xiaodong Gu, Jianbin Xiang, Kefu Liu

**Affiliations:** a School of Information Science and Technology, Fudan University, Shanghai, China; b Department of Sports Medicine, Huashan Hospital, Fudan University, Shanghai, China; c Department of General Surgery, Huashan Hospital, Fudan University, Shanghai, China; d Department of Dermatology, Huashan Hospital, Fudan University, Shanghai, China

**Keywords:** electric welding, end-to-end, *in vivo*, intestinal anastomosis, wound healing

## Abstract

To investigate the effect of high-frequency electric welding (HFEW) on intestinal tissue healing, we performed end-to-end anastomosis experiments in New Zealand rabbits. Within one week post-surgery, animals exhibited normal vital signs, replaced necrotic tissue with healthy collagen, and showed improved tissue strength while inflammation decreased. By day 60, tissue pathology and function fully recovered, resembling normal tissue. Healing at the anastomotic site occurred in three phases: immediate adhesion, inflammation, and remodeling, with macrophages crucial for phagocytosis and regeneration of necrotic tissue. This study enhances understanding of HFEW’s healing mechanisms and supports further preclinical investigations.

## Graphical abstract

**Figure:**
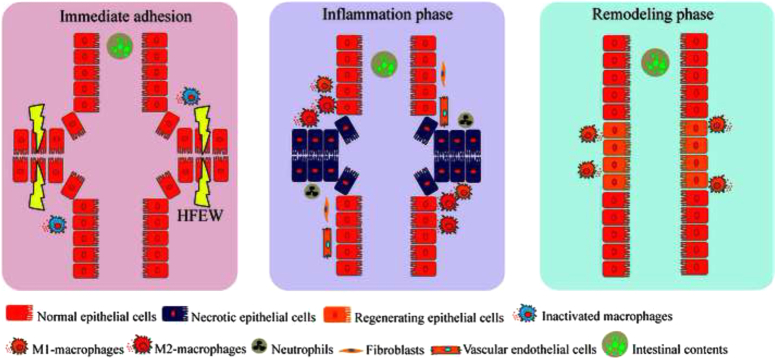

## Introduction

Highlights

- This study marks the first application of HFEW technology for intestinal end-to-end anastomosis.
- HFEW does not affect animal survival and promotes effective tissue healing, which has significant implications for the field of surgical suturing, offering a potential avenue for sutureless anastomotic devices and guiding future clinical use of HFEW.
- These findings advance our understanding of electrosurgery and have far-reaching implications for suturing techniques.

HFEW offers several advantages over traditional hand-sewn and stapler methods^1,2^, including shorter procedure times, reduced bleeding, no residual foreign bodies, and lower inflammation^3,4^. However, HFEW is still in its early stages, with several unresolved issues hindering its development^5–7^.

Post-HFEW treatment, the anastomosis site exhibits significant changes, characterized by increased fragility, hardening, and diminished tissue vitality, potentially leading to protrusions^5–7^. In-vivo experiments must address two key questions: whether necrotic tissue can temporarily occlude surrounding tissues and if it can regenerate into durable closure tissue.

To explore the healing mechanism after HFEW, we conducted end-to-end intestinal anastomosis experiments in New Zealand rabbits, assessing HFEW’s effects on vital signs, including diet, bowel activity, and mental state. We also examined microscopic changes at the anastomotic site, focusing on strength, pathology, collagen, and immune cell variations.

## Materials and methods

### Ethical approval

The experimental animals were New Zealand rabbits (*n*=40). All procedures were approved by the Ethics Committee of Shanghai Jiao Tong University (Permit Number 20200701).

### Anastomotic device

**Figure 1:**
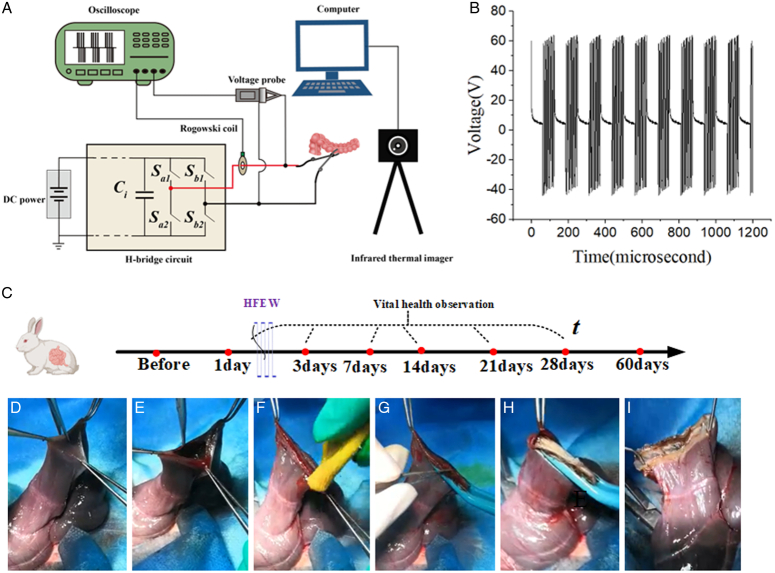
Experimental setups and surgical procedures. (A) Pulse generator circuitry and apparatus. (B) Voltage waveform for HFEW. (C) Animal post-experiment vital sign observations (1 day, 3 days, 7 days, 14 days, 21 days, 28 days). (D–H) Intestinal end-to-end anastomosis with HFEW: locate intestine, cut, clean, and anastomose. (I) Anastomotic site tissue.

The pulse generator’s primary function is to deliver square-wave pulses (440 kHz, 60 V, 5 s) to the intestinal tissue at a 75% duty cycle (Fig. 1A, B). An open technique was used for end-to-end mucosa-to-mucosa anastomosis (Fig. 1C–I) (movie). For preoperative and postoperative preparations, see the supplementary materials (SI Appendix, Methods, Supplemental Digital Content 1, http://links.lww.com/JS9/D502).

### Anastomosis strength test

Intestinal anastomotic strength testing was accomplished primarily by bursting pressure (BP) testing and thickness measurement (Supplementary Fig. S1, Supplemental Digital Content 1, http://links.lww.com/JS9/D502; Fig. S2, Supplemental Digital Content 1, http://links.lww.com/JS9/D502).

### Pathology tests

After a certain period of postoperative observation, the tissue of the surgical site was studied pathologically to investigate the healing characteristics of the anastomosis site (AS) by HE-stained sections. Simultaneous immunofluorescence analysis of tissue immune cells at the anastomosis was performed (SI Appendix, Methods, Supplemental Digital Content 1, http://links.lww.com/JS9/D502).

### Statistical analysis

Data analysis was performed using Prism 8 (GraphPad, San Diego, CA, USA).

## Results

### Characteristics of changes in vital signs

Increased temperature leads to immediate tissue adhesion at the anastomotic site due to collagen deformation but also causes thermal damage. Postoperatively, dietary intake, fecal output, body weight, and mental status sharply declined on the first day (*P*<0.05), began to rise by day 3, and returned to preoperative levels by day 5 (*P*>0.05). Additionally, burst pressure and tissue thickness fluctuated over time, with the lowest burst pressure on the first day (*P*<0.05), recovering by day 14 (*P*>0.05) (Supplementary Fig. S3, Supplemental Digital Content 1, http://links.lww.com/JS9/D502; Supplementary Fig. S4, Supplemental Digital Content 1, http://links.lww.com/JS9/D502; Supplementary Fig. S5, Supplemental Digital Content 1, http://links.lww.com/JS9/D502; Supplementary Fig. S6, Supplemental Digital Content 1, http://links.lww.com/JS9/D502).

### Postoperative changes in AS external morphology

**Figure 2:**
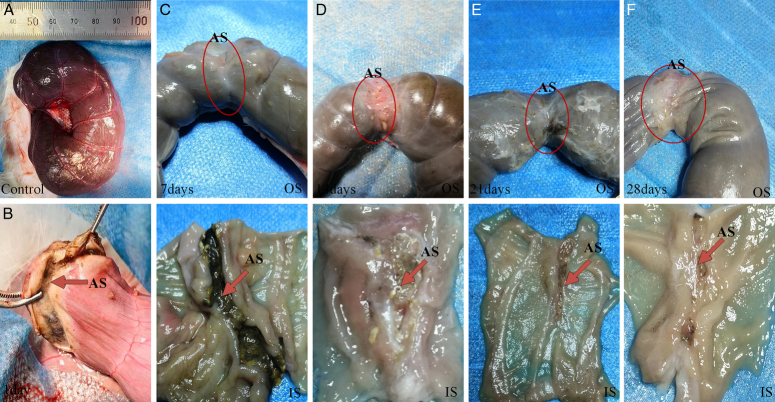
Changes of pathological characteristics of small intestine after HFEW. (A, B) Control and HFEW-treated groups at day 1. (C–F) Inner (IS) and outer (OS) surfaces of the AS at 7, 14, 21, and 28 days, with red arrows highlighting the weld seam tissue.

After HFEW treatment, the outer surface (OS) developed a compact, outwardly protruding folded structure, effectively preventing content leakage (Fig. 2A, B). On day 7, feces passed normally with no anastomotic leaks. Atrophic scars formed, and inner surface (IS) ‘grooves’ appeared dark (Fig. 2C). By day 14, tissue regeneration thickened the intestinal wall, and the outer folded structure disappeared (Fig. 2D; Supplementary Fig. S3, Supplemental Digital Content 1, http://links.lww.com/JS9/D502). Days 21 and 28 showed continued improvement, reduced scarring, thinner walls, and structural alignment with adjacent tissue (Fig. 2E, F). At day 60, the anastomosis site’s thickness resembled normal tissue, and the intestinal surface was smooth with minimal scarring (Supplementary Fig. S7, Supplemental Digital Content 1, http://links.lww.com/JS9/D502).

### Histopathological analyses of the AS

After HFEW treatment, tissues formed a tight barrier for intestinal protection (Supplementary Fig. S9, Supplemental Digital Content 1, http://links.lww.com/JS9/D502). On the first post-surgery day, compressed anastomotic tissue and unclear pathology were seen. By day 7, OS was smooth and IS displayed an incomplete trumpet shape. This shape disappeared by day 14, with muscular structures at AS. New tissue replaced necrotic tissue by days 21 and 28. By day 60, both OS and IS were smooth, and villi covered the AS mucosal layer (Supplementary Fig. S7, Supplemental Digital Content 1, http://links.lww.com/JS9/D502; Fig. S8, Supplemental Digital Content 1, http://links.lww.com/JS9/D502).

### Immunofluorescence analyses of the AS

**Figure 3:**
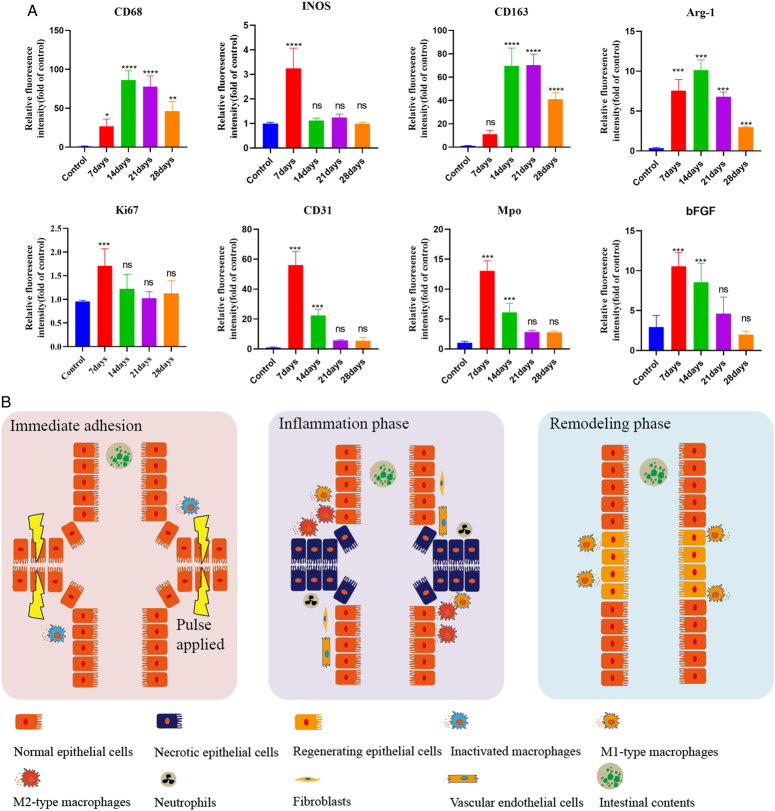
Immunofluorescent staining results of the small intestine. (A) Relative fluorescence intensity of CD68, INOS, CD163, ARG-1, ki67, CD31, MPO, and bFGF antibodies compared to the control group. Bar graphs depict mean values±SEM. (B) Schematic representation of the anastomotic tissue repair process after HFEW treatment, comprising three critical phases: immediate adhesion, inflammatory phase, and remodeling phase.

Macrophages (MPs) are vital for wound healing and tissue regeneration. We studied CD68, iNOS (M1), CD163, and Arg-1 (M2) to assess MPs in anastomosis reconstruction. CD68 fluorescence significantly increased by day 7, peaking on day 14, then declined to normal by day 60. iNOS peaked on day 7 and returned to baseline by day 28. CD163 significantly increased by day 14. Ki67 and CD31 levels rose significantly in the first week but returned to baseline by day 28, indicating rapid cell proliferation. Myeloperoxidase (MPO) and basic fibroblast growth factor (bFGF) also peaked by day 7, returning to baseline by day 28 (Fig. 3A and Supplementary Fig. S10, Supplemental Digital Content 1, http://links.lww.com/JS9/D502).

### Wound healing stage of the AS

We identified three stages in intestinal anastomotic healing: the immediate adhesion phase, the inflammatory phase (days 1–14), and the remodeling phase (days 14–60) (Fig. 3B), where tissue repair occurs and inflammatory cells decrease, ultimately resembling normal intestinal tissue by day 60.

## Discussion

HFEW presents a promising alternative. Most research on intestinal tissue welding has been *in vitro*, with limited in-vivo studies on HFEW applications^8^. This study examines physiological changes in animals after end-to-end welding with HFEW, focusing on tissue regeneration. HFEW creates immediate adhesion through thermal effects. While some thermal impacts may delay recovery, HFEW does not adversely affect healing or vital signs. Postoperatively, food intake and bowel function initially decline but improve by day 3, returning to normal by day 5.

## Conclusions

HFEW demonstrates excellent feasibility and safety for intestinal anastomosis, achieving a 100% surgical survival rate in New Zealand rabbits. Subjects returned to normal health within one week, indicating HFEW’s promising potential as a next-generation anastomosis technique.

## Ethical approval

All procedures were approved by the Ethics Committee of Shanghai Jiao Tong University. Fifty rabbits weighing 2 kg were used (Permit Number 20200701).

## Consent

As this study is a New Zealand rabbit experiment, the required Informed consent is waived.

## Source of funding

This research was supported by the National Natural Science Foundation of China (51877046) and the Pioneering Project of the Academy for Engineering and Technology of Fudan University (gyy2018-002).

## Author contribution

C.Z.: conceptualization, investigation, methodology, software, and writing – review and editing; Y.N., Z.Y., and X.C.: methodology, investigation, and software; P.X., X.T., and L.C.: methodology, investigation, and writing; J.Q., H.Z., and X.G.: methodology, supervision, and review and editing; J.X.: conceptualization, methodology, project administration, and supervision; K.L.: conceptualization, methodology, funding acquisition, project administration, supervision, and writing – review and editing.

## Conflicts of interest disclosure

The authors declare no conflicts of interest.

## Research registration unique identifying number (UIN)

Not applicable.

## Guarantor

Kefu Liu.

## Data availability statement

The data that support the findings of this study are available from the corresponding author upon reasonable request.

## Provenance and peer review

Not applicable.

## Supplementary Material
