# Supplementary material for: In-vivo tissue healing mechanism at the intestinal anastomosis site following high-frequency electric welding
Source: Int J Surg. 2024 Oct 11;111(1):1614–8. doi: 10.1097/JS9.0000000000002093 (PMC11745754; doi:10.1097/JS9.0000000000002093)
Supplement: Supplementary file 1 [file js9-111-1614-s001.docx]

Supporting Information for

In Vivo Tissue Healing Characteristics at the Intestinal Anastomosis Site Following High-Frequency Electric Welding

**This PDF file includes:**

Supporting Text: SI Methods

Figure. S1 to S10

SI References

**Methods**

**Surgical procedure**

This study involved the selection of 40 New Zealand rabbits, aged approximately 3 months, with body weights falling within the range of 1.9-2.0 kg .The treatment time and number of experimental animals are shown in Table S1. An infrared thermal imager (Fortric-200, xxx, Figure.1A) was used to monitor the temperature at the AS throughout the experiment.Within 24 hours prior to surgery, the rabbits refrained from food intake. The surgical suite and instruments underwent stringent pre-surgical sterilization procedures, with the surgical procedure being carried out by a surgeon from xxx Hospital. Preceding surgery, each rabbit received a 0.7ml anesthetic (zoletil50, VIRBAC), injection into the muscle. Subsequently, the rabbits' body weights were recorded, and blood samples were collected from the ear marginal vein. Subsequent to these preparatory steps, fur was meticulously removed from the relevant abdominal region, followed by comprehensive disinfection using iodine and 75% alcohol. An incision of approximately 3 centimeters in length was made along the abdominal midline. Thereafter, the colon was gently extracted, and the two ends of the intestinal tract were clamped using intestinal forceps. A section of the mesentery and intestine was excised. To prevent potential internal residue leakage, the intestinal lumen was carefully cleansed with moist gauze. Upon completion of the anastomosis, the intestinal tube was gently compressed to assess airtightness at the anastomotic site. In the event of gas leakage, a re-anastomosis was performed using the linear stapler. Finally, the intestine was slowly returned to the abdominal cavity, and closure of the abdominal wall was achieved via needle and thread suturing.

Table S1. Grouping information of experimental animals

| Group | Feeding time | Size of  animal | Number |
| --- | --- | --- | --- |
| Control | Day 28 | 5 | E1、E2、E3、E4、E5 |
| HFEW | Day 7 | 8 | E6、E7、E8、E9、E10、E11、E12、E13 |
|  | Day 14 | 8 | E14、E15、E16、E17、E18、E19、E20、E21 |
|  | Day 21 | 8 | E22、E23、E24、E25、E26、E27、E28、E29 |
|  | Day 28 | 8 | E30、E31、E32、E33、E34、E3、E36、E37 |
|  | Day 60 | 3 | E38、E39、40 |

**Anastomosis Strength Test**

Bursting pressure (BP) is the highest pressure recorded by the pressure gauge during infusion. The BP measurement setup consists of three main components: a peristaltic pump, a pressure sensor, and the porcine bowel specimen being tested (SI Appendix, Figure.S1). Instrument construction and test methods are similar to those documented in the literature^26^. We tested anastomotic strength in the control group and HFEW-treated groups at 7, 14, 21, and 28 days (SI Appendix, Figure.S2). We did five rounds of experiments at each time point. After BP tests, we used scissors to open the intestines, flattened the anastomosis, and measured its thickness with a vernier caliper. (SI Appendix, Figure.S2).


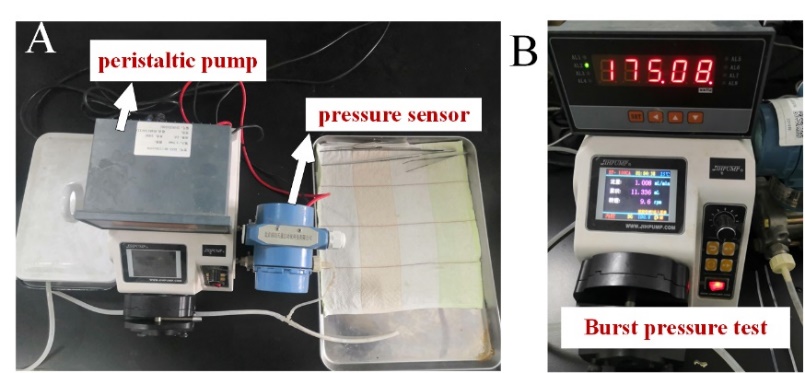


Figure.S1. Experimental setups for small intestine fusion and bursting pressure test.

A.Peristaltic pump and pressure sensor for burst pressure test. B. Burst pressure test for fused small intestine.


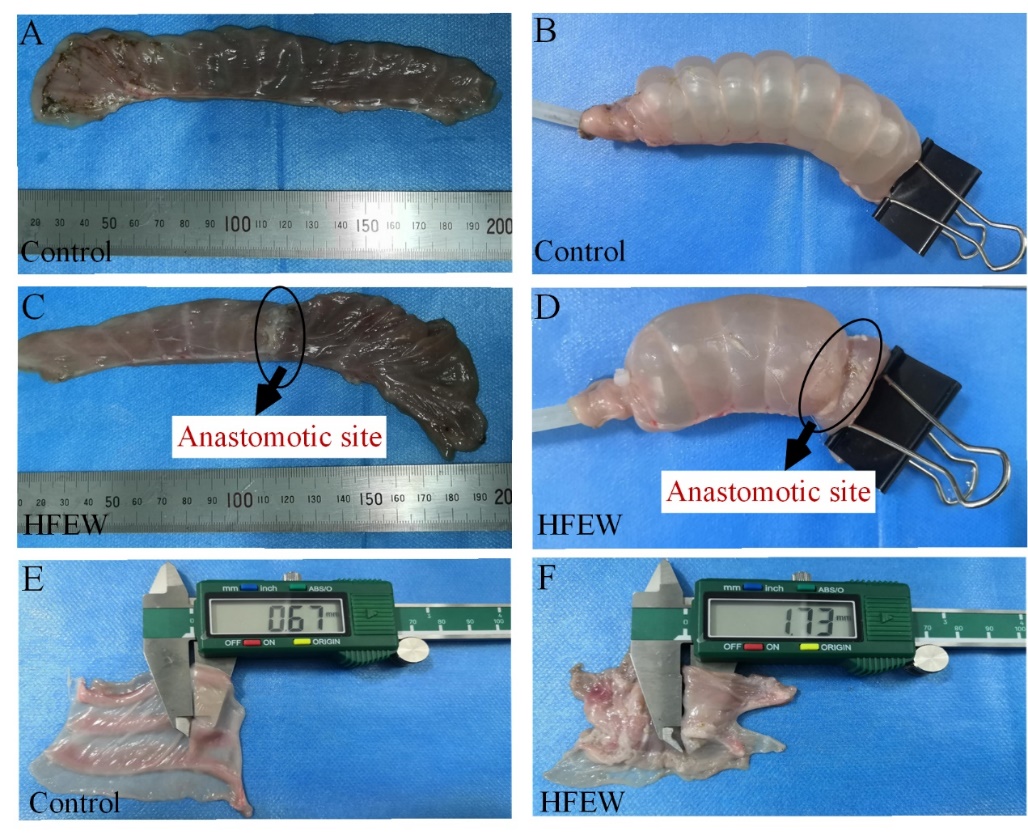


Figure.S2. Burst pressure measurement and thickness measurement at the anastomosis of samples from control group and HFEW group. A-B. burst pressure test of samples from control group and control group .C-D. burst pressure test of samples from HFEW group and HFEW samples. E-F. Sample thickness test of control group and HFEW group. The black oval is the anastomosis site.

**Postoperative period**

The surgical procedure lasted for about an hour, and within half an hour after the surgery, all animals had fully recovered from their state of unconsciousness. They were administered antibiotics daily for a total of five days post-surgery. On the 3rd, 5th, 7th, 14th, 21st, and 28th days, measurements were taken for food intake, fecal output, and the body weight of the New Zealand rabbits. Additionally, fecal samples were collected for color observation and fecal occult blood tests. Furthermore, their mental states were observed, and you can find the criteria for mental state scoring in Supplementary Figure.S5. On the 7th, 14th, 21st, 28th, and 60th days post-surgery, five New Zealand rabbits were euthanized.

**Immunofluorescence**

CD68 is a typical marker for macrophages. It assists in the identification and labeling of macrophages, which play crucial roles in the immune system, such as phagocytosis, clearance of cellular debris, defense against infections, and involvement in inflammation and immune responses^1, 2^ . The quantity of CD68 reflects the overall number of macrophages

iNOS (Inducible Nitric Oxide Synthase) is an enzyme and a marker for M1-type macrophages. Its primary function is the synthesis of nitric oxide (NO). iNOS participates in the regulation of the immune system by producing nitric oxide. Nitric oxide serves as a signaling molecule that modulates the activity of immune cells, including macrophages, lymphocytes, and granulocytes. It plays a significant role in infection, inflammation, and immune responses^2^.

CD163 is a cell surface protein primarily expressed in macrophages and monocytes, serving as a marker for M2-type macrophages. CD163 plays a significant role in the anti-inflammatory process. When cells express CD163, it can participate in immune regulation and suppress inflammatory responses. The activation of CD163 can reduce the release of inflammatory cytokines and contribute to maintaining the balance of the immune system^3^

Arginase-1 (Arg-1) is an enzyme and a marker for M2-type macrophages. In certain cells, especially in some immunosuppressive cell types, the expression of Arg-1 can promote anti-inflammatory immune responses. It can influence the activity of immune cells by regulating the metabolites produced from arginine metabolism, thereby modulating inflammatory responses. Arg-1 also participates in tissue repair and regeneration processes. Particularly under certain conditions, Arg-1 can enhance wound healing, tissue regeneration, and repair, possibly through pathways such as promoting cell proliferation and collagen synthesis^1, 3^

Ki67 is a nuclear protein typically used as a marker for cell proliferation. Its primary function is to indicate the proliferative status of cells. When cells are in the division phase (M phase), the expression level of Ki67 protein significantly increases. Therefore, Ki67 is commonly used as an indicator for measuring cell proliferation rates^4, 5^

CD31, also known as PECAM-1 (Platelet Endothelial Cell Adhesion Molecule-1), is a cell surface protein primarily found on endothelial cells (vascular endothelial cells). It serves various important functions, particularly related to the activities of the vascular system and the immune system. CD31 is a cell adhesion molecule expressed on vascular endothelial cells, contributing to cell adhesion between them. CD31 plays a crucial role in immune responses and inflammation by regulating the adhesion and migration of white blood cells through interactions with other cell surface proteins^6^.

Myeloperoxidase (MPO) is an enzyme primarily found in the granules of white blood cells, especially neutrophils, and belongs to the peroxidase enzyme family. MPO plays a bactericidal role in neutrophils. When the body is infected, neutrophils migrate to the site of infection and release MPO which can generate a potent oxidizing agent, including hypochlorous acid. These oxidizing agents play a crucial role in killing pathogens such as bacteria and fungi^7, 8^.

Basic fibroblast growth factor (bFGF) is a multifunctional protein belonging to the fibroblast growth factor family. It plays a role in promoting proliferation and differentiation in various cell types. It can stimulate the growth and reproduction of many cells, such as fibroblasts and endothelial cells, contributing to the maintenance of normal tissue growth and repair. bFGF can stimulate the proliferation and migration of endothelial cells, promoting the formation of new blood vessels, which is essential for tissue repair and regeneration.

Immunofluorescence analysis was skillfully conducted to discern the presence of macrophages, vascular endothelial cells, and fibroblasts within the anastomotic tissue. This sophisticated analysis employed antibodies such as anti-CD68, anti-iNOS, anti-CD163, anti-Arg-1, anti-ki67, anti-CD31, anti-MPO, and anti-BFGF (ab955; Abcam). Following meticulous treatment with 0.1% TritonX-100 and blocking with 5% BSA, DAPI staining and mounting with a fluorescence quencher were executed with precision. The quantification of average fluorescence intensity density from the immunohistochemical analysis was skillfully performed using ImageJ software.


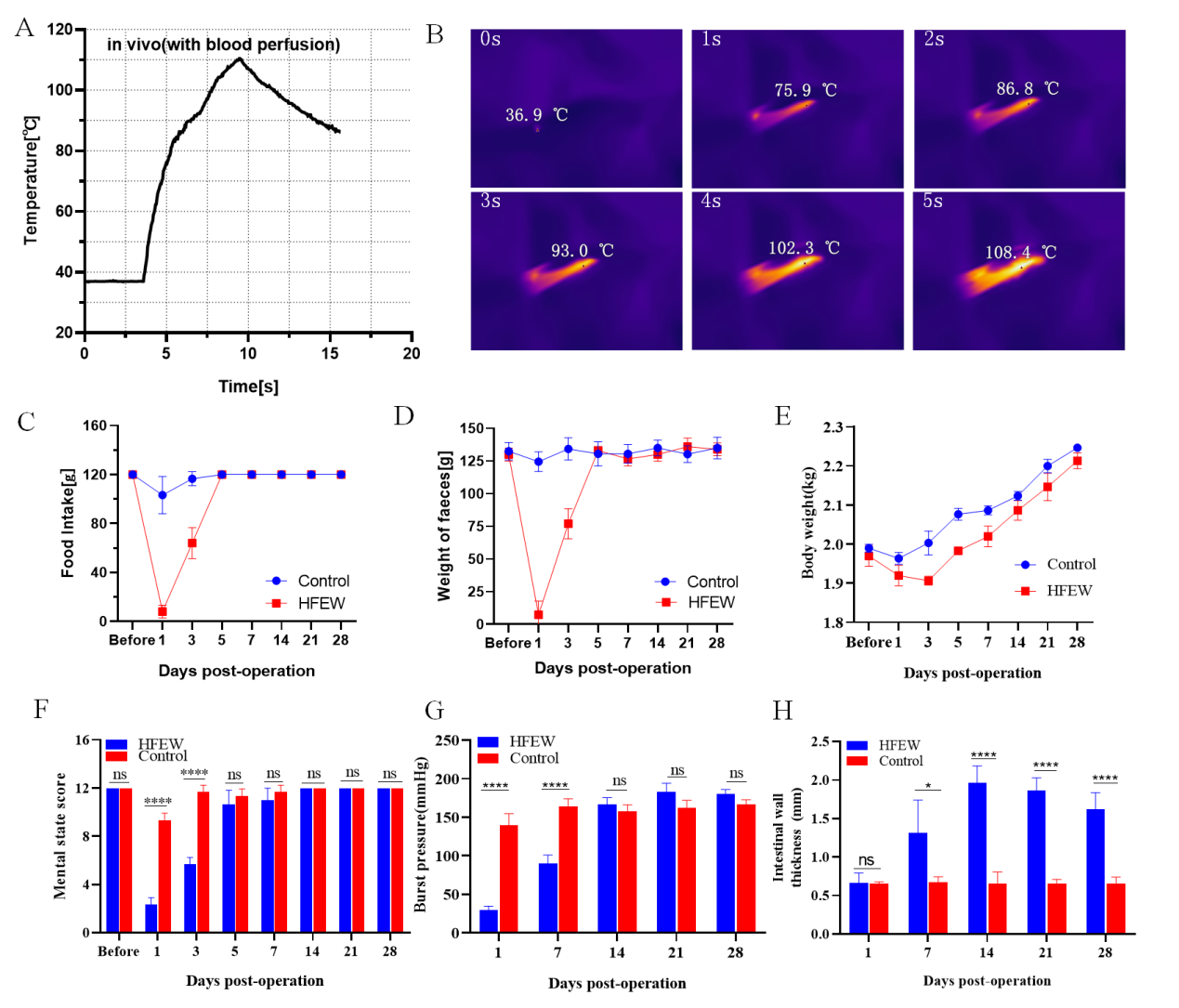


Figure.S3.Temperature detection and postoperative physiologic status assessment.A. Characterization of temperature changes over time during surgery. B. Highest temperature of AS at different time points measured with infrared imaging. C-E. Line graphs illustrating food intake, bowel movements, and weight changes over time for HFEW group and control group animals. F-H. Bar graphs depicting psychological state scores, bursting pressure, and thickness of AS changes over time for HFEW group and control group animals.


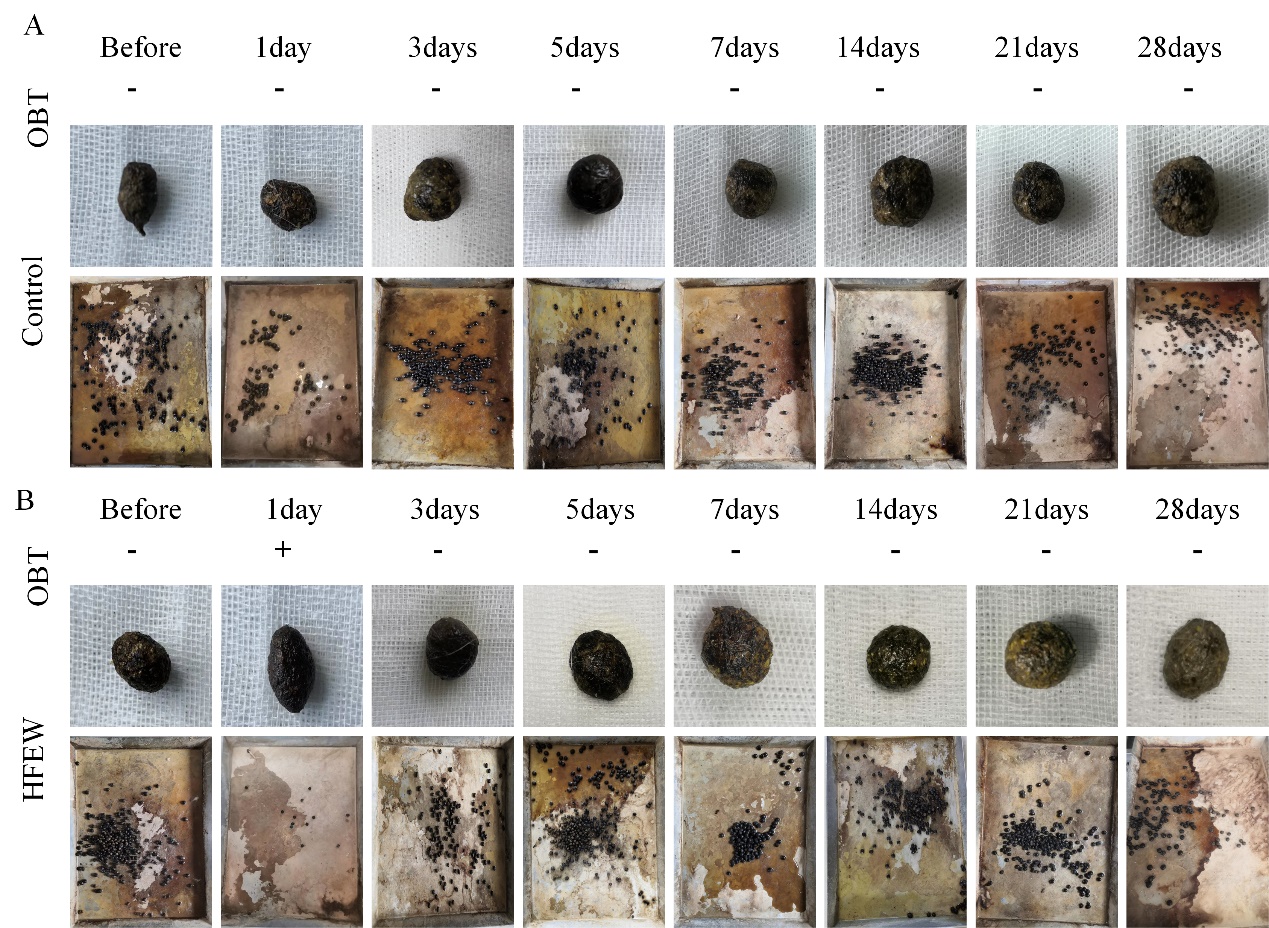


Figure.S4. Status and occult blood test results of stool samples collected in control group and HFEW group. A-B. Results of fecal status and fecal occult blood of New Zealand rabbits in control group and HFEW group. Fecal status and results of fecal occult blood of New Zealand rabbits "-" refers to negative results of fecal occult blood, and "+" refers to positive results of fecal occult blood. OBT stands for fecal Occult Blood Test.


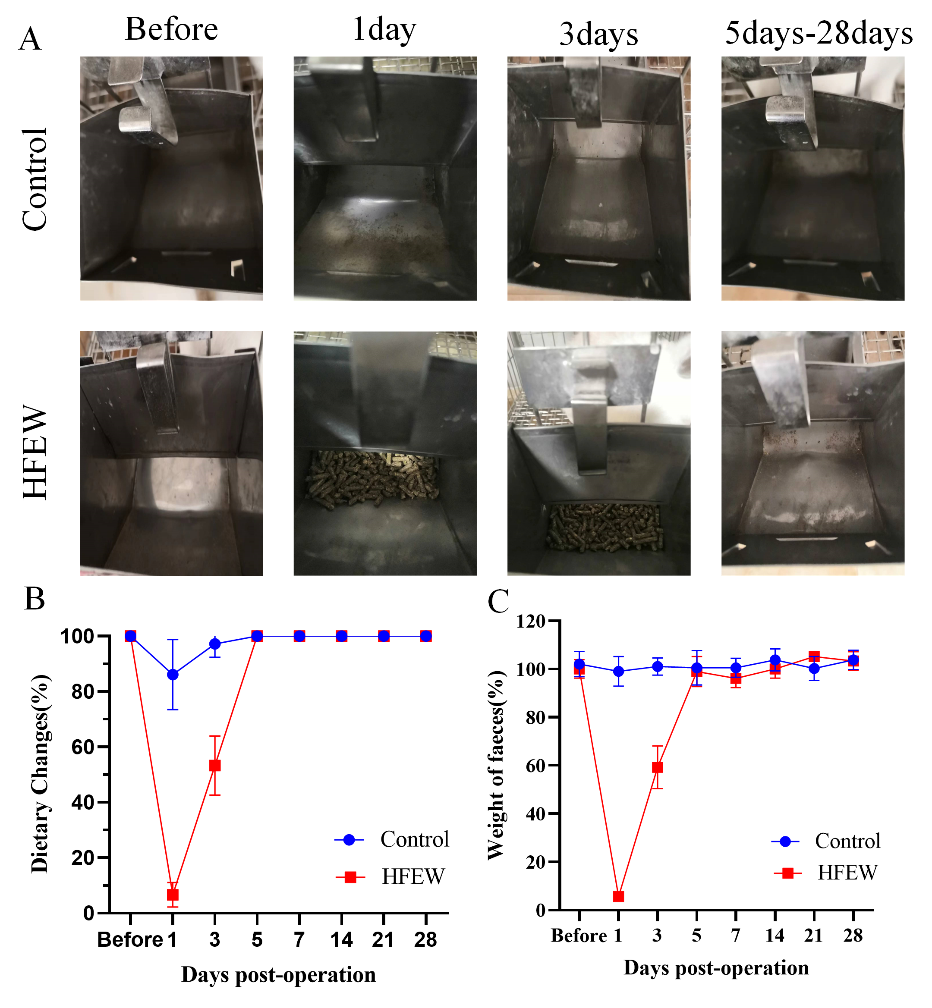


Figure.S5. Feed intake and percentage change in fecal weight of New Zealand rabbits. A. Feed intake of New Zealand rabbits. B. Percentage change in dietary volume in control group and HFEW group. C. Percentage change in fecal weight in control group and HFEW group.


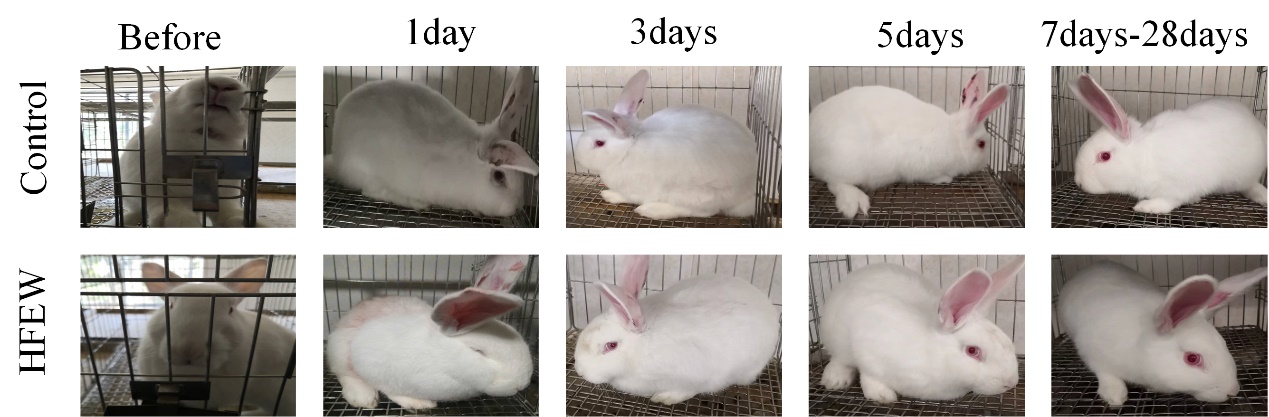


Figure.S6. Mental state evaluation of New Zealand rabbits in the control group and HFEW group after surgery. The scoring criteria are as follows: Total score: 12 points, Food intake and water consumption: 3 points, Defecation status: 3 points, Responsiveness level: 3 points, Activity level: 3 points.


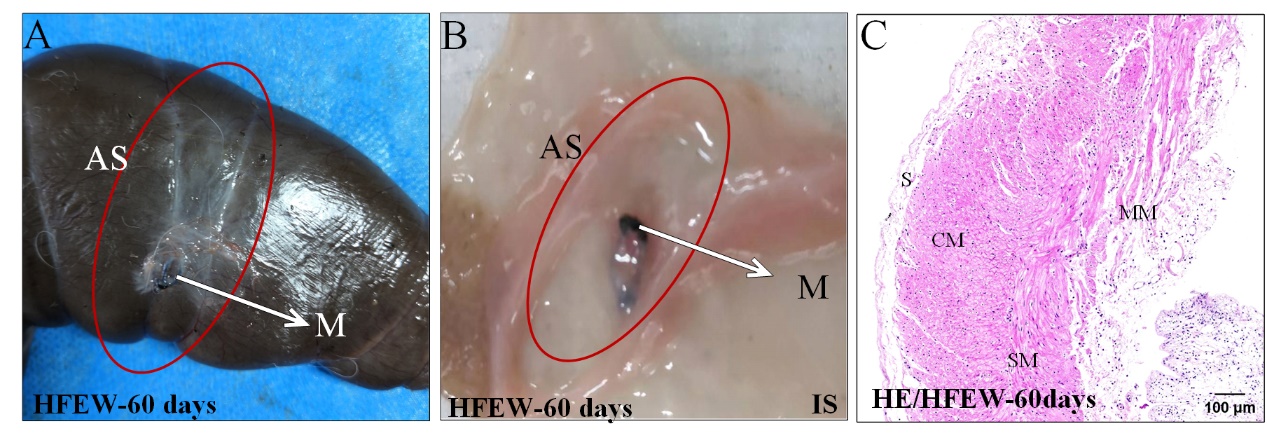


Figure.S7. Observation of the external and internal surfaces of the intestinal tract in the HFEW group at 60 days post-operation. A-B. The outer surface and inner surface of the intestine 60 days after surgery.AS refers to the anastomosing site. M refers to the marks made with non-absorbable sutures. OS refers to the initials of the outer surface.IS refers to the initials of the inner surface. C. Pathological section of the anastomosis site 60 days post-surgery.


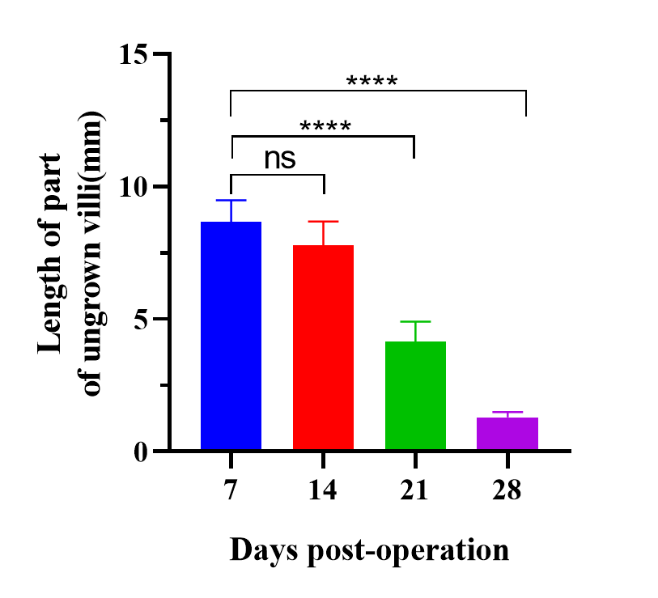


Figure.S8. Length of partially regrown villi. The villi growth of the tissues at the anastomosis was observed under the microscope and measured in the villi-free part, and the tissues at the anastomosis were all villi at 60 days postoperatively.


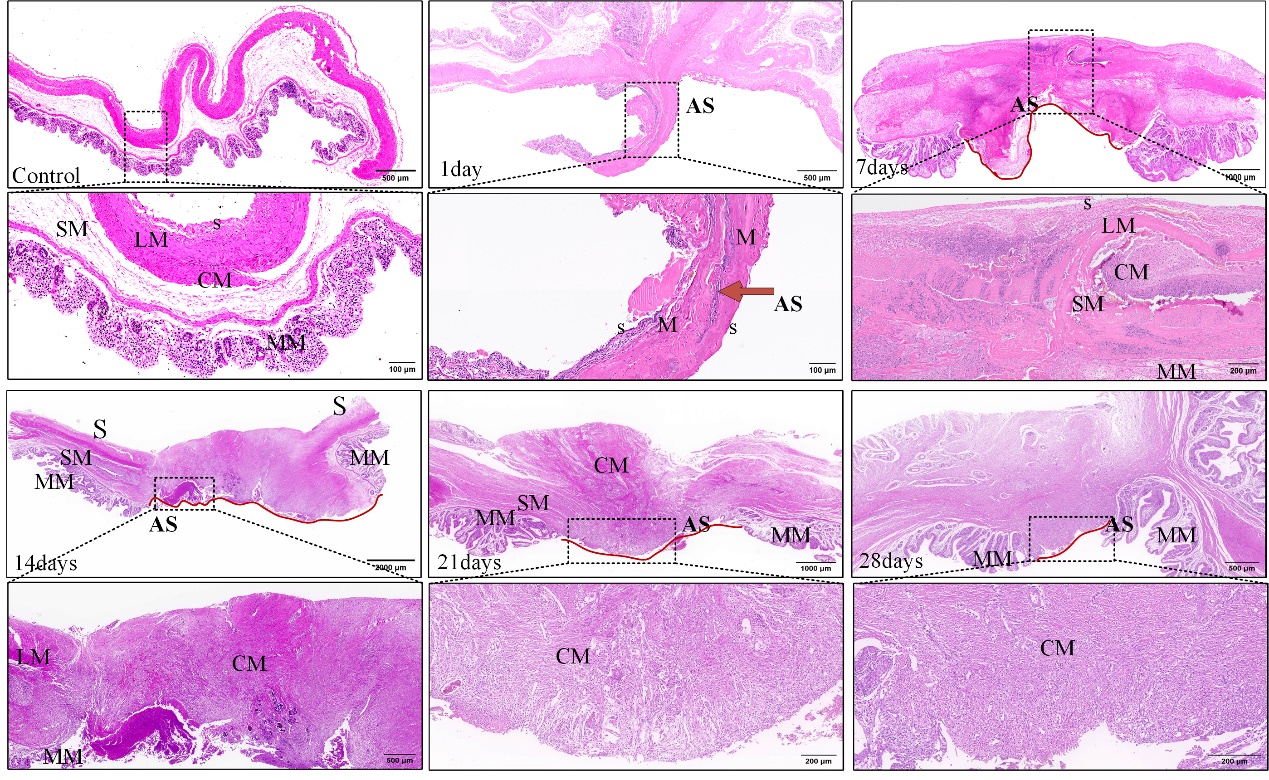


Figure.S9.H&E staining of fused small intestines on days 1, 7, 14, 21, and 28, with the magnified section shown within the black square under low magnification. MM (mucosa), SM (submucosa), CM (circular muscle), LM (longitudinal muscle), and S (serosa).


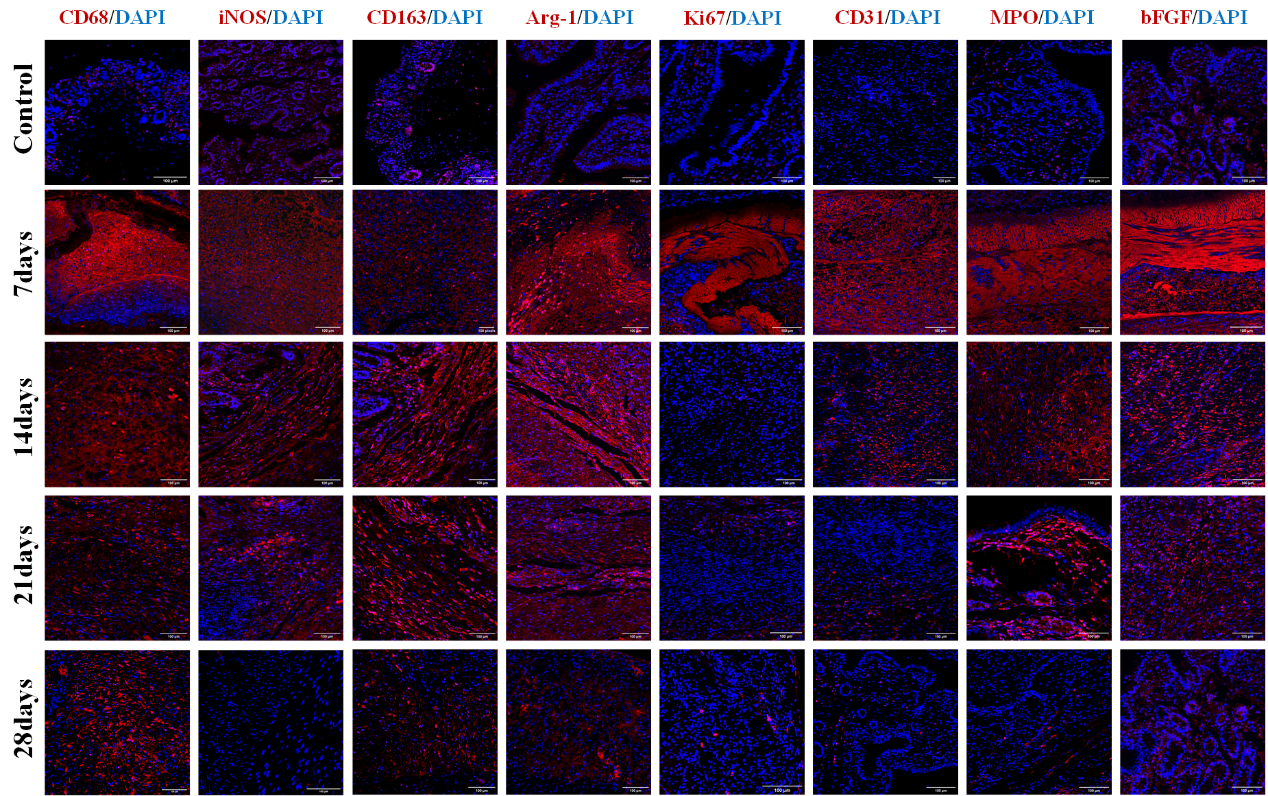


Figure. S10. Immunofluorescent staining results of small intestine.

A. Immunofluorescent double staining of intestinal tissue using antibodies CD68, INOS, CD163, ARG-1, ki67, CD31, MPO, and BFGF at D7, D14, D21, and D28 post-HFEW treatment. Blue indicates cellular nuclei stained with DAPI, while red represents CD68, INOS, CD163, ARG-1, ki67, CD31, and MPO. Scale bar = 100μm.


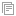


References

1. Novak, M.L. & Koh, T.J. Macrophage phenotypes during tissue repair. *Journal of Leukocyte Biology* **93**, 875-881 (2013).

2. Yang, Y. et al. Macrophage polarization in experimental and clinical choroidal neovascularization. *Scientific Reports* **6** (2016).

3. Atri, C., Guerfali, F. & Laouini, D. Role of Human Macrophage Polarization in Inflammation during Infectious Diseases. *International Journal of Molecular Sciences* **19** (2018).

4. Sun, X. & Kaufman, P.D. Ki-67: more than a proliferation marker. *Chromosoma* **127**, 175-186 (2018).

5. Zhou, Y. et al. Ki67 is a biological marker of malignant risk of gastrointestinal stromal tumors. *Medicine* **96** (2017).

6. Caligiuri, G. Mechanotransduction, immunoregulation, and metabolic functions of CD31 in cardiovascular pathophysiology. *Cardiovascular Research* **115**, 1425-1434 (2019).

7. Aratani, Y. Myeloperoxidase: Its role for host defense, inflammation, and neutrophil function. *Archives of Biochemistry and Biophysics* **640**, 47-52 (2018).

8. Davies, M.J. & Hawkins, C.L. The Role of Myeloperoxidase in Biomolecule Modification, Chronic Inflammation, and Disease. *Antioxidants & Redox Signaling* **32**, 957-981 (2020).
